# Chitosan Nanoparticles as Bioactive Vehicles for Textile Dyeing: A Proof of Concept

**DOI:** 10.3390/polym14224821

**Published:** 2022-11-09

**Authors:** Eduardo M. Costa, Sara Silva, Manuela Machado, Sérgio C. Sousa, Freni K. Tavaria, Manuela Pintado

**Affiliations:** Universidade Católica Portuguesa, CBQF—Centro de Biotecnologia e Química Fina, Laboratório Associado, Escola Superior de Biotecnologia, Rua Diogo Botelho 1327, 4169-005 Porto, Portugal

**Keywords:** textile dyeing, functionalization, chitosan nanoparticles, biocompatibility, antimicrobial cotton, nanoencapsulated dye

## Abstract

In recent years bioactive textiles have risen to the forefront of consumers perception due to their potential protection against virus, fungi and bacteria. However, traditional textile staining is an eco-damaging process that and current methods of textile functionalization are expensive, complicated and with great environmental impact. With that in mind, this work sought to show a possible solution for this problematic through the usage of a novel one step textile dyeing and functionalization method based upon nanoencapsulated textile dyes (NTDs). To do so navy blue everzol NTDs were produced with chitosan, cotton dyed, characterized through FTIR and SEM and biological potential evaluated through biocompatibility screening and antimicrobial activity against skin pathogens. The data obtained showed that NTDs effectively dyed the target textile through a coating of the cotton fibre and that NTDs formed hydrogen bonds with the cellulose fibre via electrostatic interactions of the chitosan amino groups with cotton sulphate groups. From a biocompatibility perspective NTDs dyed cotton had no deleterious effects upon a skin cell line, as it promoted cellular metabolism of HaCat cells, while traditionally died cotton reduced it by 10%. Last but not least, NTDs dyed cotton showed significant antimicrobial activity as it reduced viable counts of MRSA, MSSA and *A. baumannii* between 1 and 2 log of CFU while traditional dyed cotton had no antimicrobial activity. Considering these results the novel method proposed shows is a viable and ecological alternative for the development of antimicrobial textiles with potential biomedical applications.

## 1. Introduction

The textile industry in developed countries is confronting the world’s marketing conditions and competitive challenges, which are driving the development of advanced, highly functional textiles and textiles with high sustainability and low environmental impact. The actual usage of polymer and textiles has a vast number of advantages and attractiveness as a material. However, conventional textile finishing techniques are quite hazardous as chemicals are used in large quantities and the produced wastewaters need to be cleansed before discharging. Textile fabrics must undergo a variety of treatments before dying and printing, resulting in an increase in waste production (dyeing 1 kg of cotton requires 100 to 150 L of water and 0.6 kg of NaCl) and higher energy consumption thus creating a problem that has been on the forefront of the industry worldwide [1,2,3]. Traditional approaches for the management of this problem have been through effluent treatment with the textile industry focusing on means to remediate the polluted wastewaters [4]. However, effluent treatment is not an efficient solution and as such researchers are searching for new approaches to ameliorate this problem [5,6]. One of the less ventured approaches is the modification/alteration of the dyeing process, with existing works focusing mainly on reducing the salt and alkali content required for dyeing [7,8] or even as recently shown by Mehdi, Hussain [9], water and salts free dyeing methods using alternative solvents. One of the alternatives that has risen in later years is chitosan.

Chitosan is a polycationic polysaccharide and one of the most promising biopolymers as it possesses no toxicity and due to its high biocompatibility and biodegradability has been approved by the FDA for topical applications [10]. Additionally, the presence of amino groups in chitosan’s structure confers it high biological activity and reactivity which makes it and its derivatives, such as nanoparticles, prime candidates for textile functionalization [11,12]. In fact, in later years chitosan and its nanoparticles (NPs) have been described as being technologically viable alternatives to traditional textile finishing agents as they are capable of granting textiles antimicrobial activity, making them shrink-proof and enhanced breaking strength, wrinkle-resistance and even textile dyeing [11,13,14,15]. Between chitosan and its NPs, the latter have proven themselves to be the better candidate for particular interest as they have a high loading capacity and ability to limit compounds’ interaction with the external environment leading to reduced compound-mediated toxicity [16,17]. Recent proof of this potential has been given in a previous work by Costa, Silva [10] which showed that chitosan NPs were capable of cotton dyeing with disperse and reactive dyes without any chemical adjuvant or salt in the reaction.

Considering all of the above, this work sought to show for the first time the potential of a novel chitosan NPs-based chemical and salt-free textile dying method for the development of bioactive textiles. This premise was based upon the hypothesis that the previously described [10] nanoencapsulated textile dyes (NTDs) dyeing method was capable of in one single-step dyeing and functionalizing textiles without any chemical adjuvant and salt addition. To do so, cotton was selected as a model fabric and NTDs dyed cotton was evaluated for its biocompatibility and antimicrobial activity. Additionally, this work also sought to understand the dyeing mechanics of the proposed methodology through Fourier transform infrared spectrometer (FTIR) and scanning electron microscopy (SEM) analysis.

## 2. Materials and Methods

### 2.1. Sources of Chemicals and Solutions Preparation

Low molecular weight chitosan (LMW) was obtained from Sigma-Aldrich (St. Louis, MO, USA) and presented a deacetylation degree (DD) between 75 and 85% and a molecular weight (MW) of 107 kDa. Sodium Tripolyphosphate (TPP). Navy blue everzol (a reactive dye traditionally used for dyeing natural cellulose fibers), cotton and navy blue everzol dyed cotton using traditional methods were kindly donated by Aquitex S.A. and was prepared using ultra-pure water (Millipore SIM FILTER, Burlington, MA, USA) and stirred until complete dissolution.

### 2.2. Nanoencapsulated Dyes Production and Textile Dying

Navy blue everzol NTDs production and textile dyeing were performed as previously described by Costa, Silva [10]. Briefly, LMW chitosan was dissolved at 2 mg/mL in acetic acid that was 1.75× more concentrated and the pH value was adjusted to 5 with NaOH. TPP was used at chitosan to TPP relation of 7:1. Navy blue everzol was used at 25 mg/mL dissolved in deionized water. The process began with the addition of 4 mL of chitosan, placed under stirring at 500 rpm, to which a mixture of dye and TPP (1 and 2 mL, respectively) were gently added dropwise, at room temperature. The produced nanoparticles were shown to have an average size of 350 nm with a polydispersity index of 11.22 nm and a zeta potential of +32.7 ± 3.2 mV through dynamic light scattering (DLS, Malvern Instruments NanoZSP (Worcestershire, UK)). Following this, cotton disks were dyed through the pad-dry-cure procedure as described by Costa, Silva [10]. 

### 2.3. FTIR-ATR Analysis

The spectra of cotton and dyed cotton were obtained with a FTIR (PerkinElmer Spectrum-100), with a horizontal attenuated total reflectance (ATR) accessory, with a diamond/ZnSe crystal. All spectra were acquired with 128 scans and 32 cm^−1^ resolution, in the region of 4500 to 450 cm^−1^. Three replicates were collected for each sample.

### 2.4. Surface Evaluation by Scanning Electron Microscopy

Dyed textile surface morphology was evaluated by scanning electron microscopy using a JSM-5600LV microscope (from JEOL, Tokyo, Japan). For SEM observation, the samples were placed on top of double-sided adhesive carbon tape (NEM tape, from Nisshin, Japan), and coated with gold/palladium, using a Sputter Coater (from Polaron, Bad Schwalbach, Germany). SEM was operated in high-vacuum mode, using a spot size of 16–30 and an accelerating voltage of 18–30 kV.

### 2.5. Biocompatibility Evaluation

#### 2.5.1. Cell Line Growth Conditions

Human keratinocyte cell line (HaCat) was obtained from Cell Line Services (Appenheim, Denmark). The cells were cultured, at 37 °C in a humidified atmosphere of 95% air and 5% CO_2_, as monolayers using Dulbecco’s Modified Eagle’s Medium (DMEM) with 4.5 g/L glucose, L-glutamine without pyruvate (Lonza, Verviers, Belgium) containing 10% (*v/v*) fetal bovine serum (FBS, Biowest, Nuaillé, France) and 1% (*v/v*) Penicillin-Streptomycin-Fungizone (Lonza, Verviers, Belgium).

#### 2.5.2. Biocompatibility Assays

Dyed cotton biocompatibility was assayed through adaptation of the procedure previously described by Maryan, Montazer [18]. Briefly, HaCat cells were seeded in the wells of a 96 well microplate and allowed to adhere for 24 h. Simultaneously, cotton fabrics (non-dyed, with navy blue everzol and with nanodye) were cut into 1 × 1 cm pieces, which were then soaked in 2 mL of DMEM for 24 h. After 24 h the media was removed, and the cells were washed with PBS. Following this media with leached substances (direct and 1/2 diluted) was added. After 24 h, 25 µL of XTT working solution were added to each well and the cells were incubated, in the dark, for 2 h. The optical density (OD) at 485 nm was then measured using a microplate reader (FLUOstar, OPTIMA, BMG Labtech, Ortenberg, Germany). All assays were performed in quintuplicate.

### 2.6. Antimicrobial Activity

#### 2.6.1. Microorganisms

*Acinetobacter baumannii* (*A. baumannii*) and methicillin-resistant *Staphylococcus aureus* (MRSA) were obtained from the culture collection of the Göteburg University (CCUG) (Sweden) (CCUG 61012). Methicillin-sensitive *Staphylococcus aureus* (MSSA) was obtained from American Type Culture Collection (ATCC 25923).

#### 2.6.2. Antimicrobial Activity

The antimicrobial activity of the impregnated cotton disks was performed accordingly to the ISO 20743 standard [19]. Briefly, inocula were grown overnight, at 37 °C, in Tryptic Soy Broth (Biokar Diagnostics, Beauvais, France) after which the bacterial load was adjusted to a concentration between 1 × 10^5^ to 3 × 10^5^ CFU/mL. Following this, 200 µL of adjusted inoculum was added to 0.40 g (± 0.05 g) of cotton dyed with blue navy everzol NPs (25 mg/mL). Simultaneously, two controls were accessed; one with non-dyed cotton the other with cotton dyed with navy blue everzol at 25 mg/mL. Viable counts were determined at 0 and 24 h and plated by the drop method as previously described by Costa, Silva [20] in Plate Count Agar (Biokar Diagnostics, Beauvais, France). Plates were then incubated at 37 °C for 24 h and the results were given in log of CFU. All assays were done in sextuplicate.

### 2.7. Statistical Analysis

Statistical analysis was performed using IBM SPSS Statistics v21.0.0 (New York, NY, USA) software. As the data followed a normal distribution, One-way ANOVA coupled with Turkey’s post hoc test was used to assess the differences between the results observed with differences being considered significant for *p*-values below 0.05.

## 3. Results

### 3.1. FTIR-ATR

**Figure 1:**
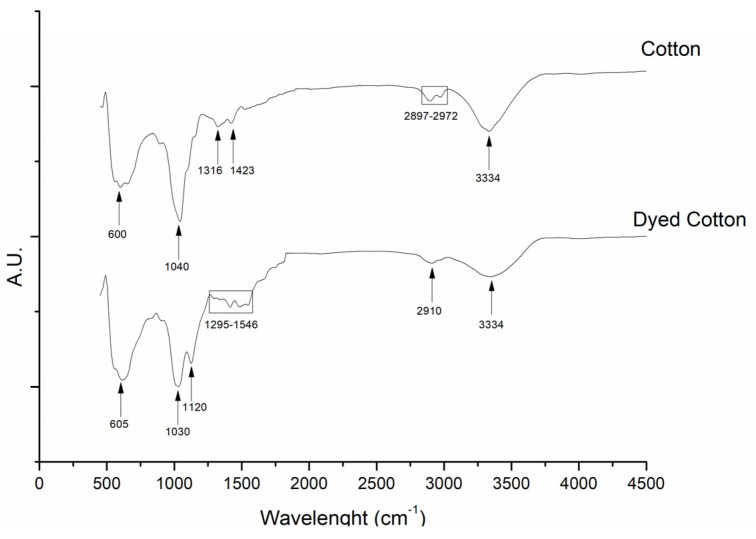
FTIR characterization of cotton dyed with nanoencapsulated navy blue everzol.

As can be seen in Figure 1 the characteristic peaks of cotton can be seen in the blank cotton with the peaks at 3334 cm^−1^ representing the cellulose molecule intra-molecular hydrogen bonding, the peaks between 2972 and 2897 cm^−1^ representing the CH_2_ symmetrical and asymmetrical stretching, the peaks at 1423 and 1316 cm^−1^ represent, respectively, the CH_2_ scissoring and rocking of the cellulose carbon structure, the peak at 1040 cm^−1^ represents the C-O stretch and the peaks at 890 and 600 cm^−1^ represent, respectively, the β-linkage of cellulose and the OH out-of-plane bending.

When considering the FTIR spectra obtained for the cotton dyed with navy blue everzol NTDs 12 peaks could be observed, of which 7 were characteristic of cotton and 5 characteristic of chitosan NPs. The cotton characteristic peaks identified were the ones at 334 cm^−1^, which represented the cellulose molecule O-H groups intra-molecular hydrogen bonding, the ones at 1411 and 1338 cm^−1^, which represent the CH_2_ scissoring and rocking of the cellulose carbon structure, the one at 1295 cm^−1^, which may represent the C=O stretching or the NH_2_ deformation of the cellulose structure, and the peak 600 cm^−1^ which represents and the OH out-of-plane bending. On the other hand, the NPs characteristic peaks identified were the one at 2910 cm^−1^, which represents the CH_2_ vibrations of the pyranose ring, the ones at 1546 and 1485 cm^−1^, which represent the NH_2_ deformation and the CH_2_ bending of NPs, and the ones at 1120 and 1026 cm^−1^ which represent the P=O stretching in NPs and the COH stretching and C-N vibrations.

### 3.2. Surface Evaluation by SEM

**Figure 2:**
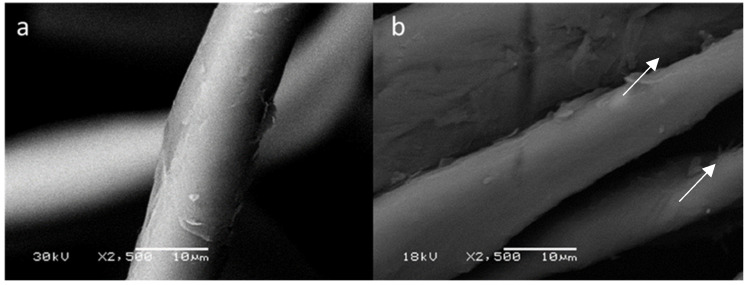
FTIR characterization of cotton dyed with nanoencapsulated navy blue everzol. (**a**)undyed cotton. (**b**) NTDs dyed cotton. Arrows show coated areas of the fiber.

When comparing the SEM imaging (Figure 2) results obtained for cotton and NTDs dyed cotton it is possible to see that navy blue everzol NTDs dyed the fabrics through a coating of the individual fibers, as a layer of NTDs over the surface can be observed in Figure 2b. This was particularly evident at the filament crossings where accumulations of NTDs can be seen (white arrows).

### 3.3. Biocompatibility Assays

**Figure 3:**
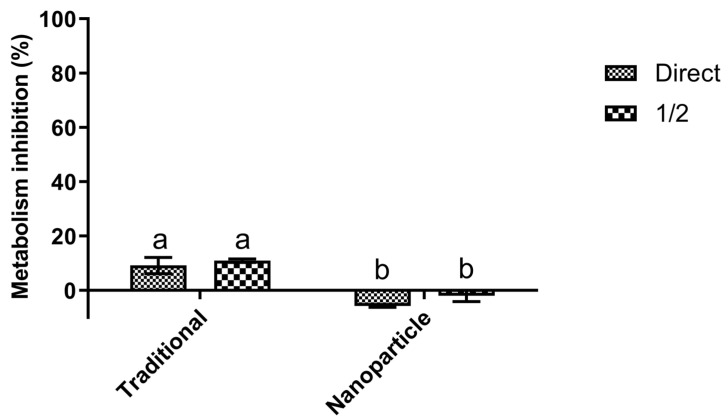
HaCat biocompatibility of cotton dyed traditionally and with navy blue everzol NTDs. Different letters represent statistically significant differences found (*p* < 0.05) for each assay. Different letters represent the differences found (*p* < 0.01) between the tested conditions.

The textile biocompatibility results obtained can be observed in Figure 3. Analysis of the results showed NTDs dyed cotton was not cytotoxic towards HaCat cells while traditionally dyed cotton presented statistically significant higher (*p* < 0.01) HaCat metabolism inhibition values, with no concentration-related effect being observed.

### 3.4. Antimicrobial Activity Assays

**Figure 4:**
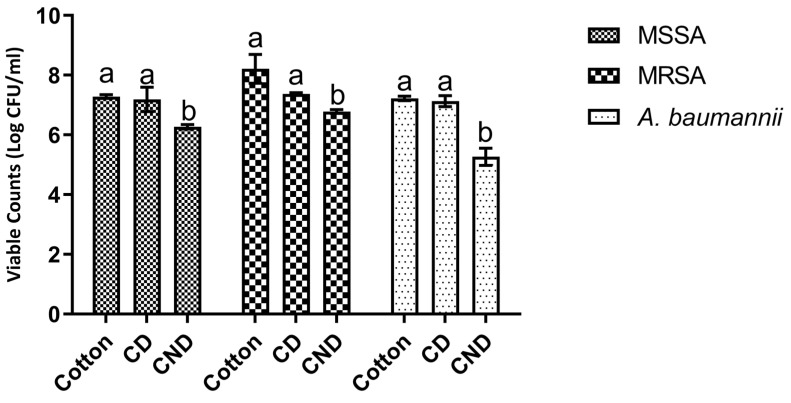
Antimicrobial activity results of cotton dyed traditionally and with navy blue everzol NTDs obtained by viable cell enumeration. All values, in log CFU/mL, corresponding to the average of 6 replicates. Different letters represent statistically significant differences found (*p* < 0.05). CD—traditionally dyed cotton; CND—NTDs dyed cotton.

When analysing the results obtained it is possible to see (Figure 4) that for all microorganisms tested NTDs dyed cotton had higher antimicrobial activity than traditionally dyed cotton with statistically significant (*p* < 0.05) lower viable counts being obtained.

Additionally, it is interesting to note that for all tested microorganisms traditionally dyed cotton did not present any statistically significant (*p* > 0.05) reductions of viable counts relative to the undyed control. In fact, except for MRSA, viable counts registered for both controls (undyed and traditional) were virtually equal. When considering the NTDs dyed cotton the more antibiotic-resistant microorganisms tested presented the highest reductions, as reductions of 1, 1.43 and 2 log of CFU were registered for MSSA, MRSA and *A. baumannii* respectively.

## 4. Discussion

Chitosan and its NPs are widely described in the literature as enhancers of traditional textile dyeing techniques and as previously seen in the conjugation of the nanoencapsulation process with the pad-dry-cure technique possess potential as an environmentally friendly alternative for textile dyeing [10,21]. However, as no previous works exist describing the incorporation of chitosan NTDs into cotton, comparisons must be drawn against void chitosan NPs and traditional dyeing technologies when possible.

When regarding the FTIR results of the NTDs dyed cotton the spectra obtained is similar to those previously reported in the literature with the successful incorporation of the chitosan NTDs into cotton being characterized by the presence of amino groups in the spectra of the dyed textile [22]. Another indicator of the successful incorporation of the navy blue everzol NTDs into cotton was the broadening and weakening of the band at 3334 cm^−1^, which represents the cellulose intra-molecular hydrogen bonding in cotton, from cotton to NTDs dyed cotton, similar to what was described by Wang, She [23] and by Hasanin, Swielam [24]. These alterations align themselves with the void chitosan NPs-cotton mechanism previously proposed by Wu, Yu [25], De Mesquita, Donnici [26] and Tawfik and El-Masry [27], which states that void chitosan NPs presence leads to the breaking of the intermolecular hydrogen bonds of cellulose and into the formation of cellulose–chitosan hydrogen bonds and that the displacement of bands is attributed to the electrostatic interactions between the positively charged amino groups of chitosan and the negatively charged sulphate groups of the fiber surface.

SEM imaging of the fiber surface showed a smooth surface for cotton and a coated surface for NTDs dyed cotton. This is similar to the findings for void chitosan NPs of AbdElhady [28], Raza and Anwar [22], Wang, She [23] and Gadkari, Ali [29], which observed that after void NPs treatment cotton fibers presented NPs adsorbed to their surface and that NPs lost their spherical shape adopting a flat structure (as seen here) when adsorbed to the textile fiber and conferring a smoother texture to the fiber surface, as previously described [30].

With the increase in textile functionalization, as an answer to ever-rising consumer demands, textile biocompatibility has become paramount, as different chemicals and textile finishing agents may lead to a loss of biocompatibility of textile fabrics [31,32]. As shown in a previous work dye loaded NTDs on their own, and contrary to the free dyes, are biocompatible with the HaCat cell line [10]. Similarly, cotton disks dyed with navy blue everzol NTDs were biocompatible with HaCat cells while a small cytotoxic effect was observed for the traditionally dyed cotton. The biocompatibility observed for the NTDs dyed cotton is in line with that reported in various works for chitosan and void chitosan nanoparticles finishes. Chandrasekar, Vijayakumar [31] showed that void chitosan nanocomposite finishes of textiles caused no hyperaemia, haemorrhage, and coagulation, thus making them biocompatible; Tseng, Hsu [33] showed that nylon fabrics leaching products treated with chitosan had no cytotoxicity towards L929 fibroblasts, Petkova, Francesko [34] reported that cotton with a chitosan nanocoating had no cytotoxic effects towards skin fibroblasts and Alminderej, Ammar [32] showed that cotton treated with chitosan was not cytotoxic towards the Hep G2 cell line. On the other hand, François, Adeline [35] showed that polystyrene treated with chitosan led to a drop in L132 cells viability, probably due to the chitosan carboxylic acids present in the fibers. Lastly, the slight toxicity observed for the traditionally dyed cotton leaching products comes in line with the work of Bierhalz and Moraes [36], which stated that cotton threads were toxic towards human fibroblasts due to the compound used to dye them and with the various works which previously showed that reactive dyes had cytotoxic properties [37,38,39,40,41]. 

Antimicrobial functionalization of textiles is one of the most common finishing treatments applied to textiles nowadays, as the textile industry strived to answer consumer demands and produce products with added value [42,43,44]. Chitosan and chitosan-based NPs have garnered attention due to their textile applications and their usage as a finishing agent capable of imprinting antibacterial activity to fabrics [45,46,47]. However, and to the best of our knowledge, chitosan NPs have only been used as a finishing agent and not as a bioactive vehicle for textile dyeing. As such, comparisons of the dyed cotton’s antimicrobial activity must be drawn against works in which NPs were used as finishing agents. The antimicrobial activity results here reported for NTDs dyed cotton are similar to the ones in works previously published dealing with NPs functionalized cotton. Hebeish, Sharaf [21], Shahid ul and Butola [48], Joshi, Khanna [49] and Raza and Anwar [22] showed that functionalized cotton reduced *Escherichia coli* (E. coli), *Micrococcus luteus* (M. luteus) and *S. aureus* growth. Similarly, Ali, Rajendran [47] showed that polyester functionalized with chitosan NPs reduced S. aureus growth by 90% a value which is in line with the ones obtained in this work as growth was reduced by 97.84 ± 0.30% and 90.00 ± 1.66% for MRSA and MSSA, respectively. When considering loaded, or hybrid NPs, previous results fall in line with the activity here observed as AbdElhady [28] showed that cotton swathes treated with chitosan zinc oxide NPs inhibited E. coli and S. aureus growth. El.Shafei and Abou-Okeil [50] reported that cotton treated with ZnO/carboxymethyl chitosan nanocomposite also inhibited E. coli and S. aureus growth and Perelshtein, Ruderman [51] reported that cotton coated with chitosan-Zn NPs reduced E. coli and Enterococcus faecalis activity. Similarly, Farouk, Moussa [52] showed that cotton and a cotton/polyester blend impregnated with ZnO-chitosan NPs were capable of significantly reducing *E. coli* and *M. luteus* survival. However, some caution must be exercised as in these studies the antimicrobial activity cannot be attributed only to the chitosan NPs as zinc, under the right conditions, is known to possess antimicrobial potential. More recently, Arenas-Chávez, de Hollanda [53] showed that cotton functionalized with a nanocomposite of chitosan and silver had antimicrobial activity against *E. coli* and *S. aureus*, but with most of the antimicrobial activity being attributed to the zinc constituent of the nanocomposite. Similarly, Saleh, Khaffaga [54] reported that cotton treated with a chitosan-copper nanocomposites had antimicrobial activity against *S. aureus* and *E. coli* with the antimicrobial activity being attributed to the nanocomposite has a whole.

## 5. Conclusions

Overall, the results obtained showed the potential of this novel one-step green chitosan nanoparticle based dyeing method for the development of dyed cotton fibers with potential for biomedical related applications. The navy blue everzol NTDs dyed cotton presented significant antimicrobial activity against MRSA, MSSA and *A. baumannii*, without requiring any additional functional finishing and had no deleterious effect towards a skin cell line metabolism, contrary to cotton traditionally dyed. Despite stability assays being necessary to further understand the full potential of this environment friendly dyeing technique, these results prove the successful development of an easy, environmentally friendly process that allows cotton to be dyed and functionalized in one step and to be used in future biomedical textiles.

## Data Availability

The data presented in this study are available on request from the corresponding author. The data is not publicly available due to confidentiality agreements.
